# Primary Solitary Hydatid Cyst of Brain in a 12-Year-Old Boy: A Case Report

**Published:** 2019

**Authors:** Naeem RAVANBAKHSH, Navid RABIEE, Jalal AHMADI

**Affiliations:** 1. Student Research Committee, Birjand University of Medical Sciences, Birjand, Iran; 2. Department of Neurosurgery, Imam Reza Hospital, Birjand University of Medical Sciences, Birjand, Iran

**Keywords:** Echinococcosis, Brain, Cysts, Iran

## Abstract

Hydatidosis is a zoonotic disease caused by *Echinococcus* parasite that frequently involves liver and lungs. Primary intracranial hydatidosis is a rare condition which can be life threatening if ruptured. Here we report an unusual case of primary intracranial hydatid cyst without any other organ involvement, diagnosed in a 12-year-old boy in Emam Reza hospital, Birjand, Iran in November 2016, in order to focus on the importance of proper diagnosis and management, especially in endemic areas.

## Introduction

Hydatidosis is a global zoonotic disease caused by larval stage of tapeworm *Echinococcus*. *E. granulosus* strain is the most common type of infection whereas *E. multilocularis* is less common but more invasive (1, 2).

Dogs are final host and sheep are intermediate hosts. Human can also get infected as an accidental host by ingestion of food, milk or water contaminated by ova of the parasite. Hydatidosis is endemic in Australia, New Zealand, Mediterranean countries, the Middle East and South America. Iran is an important endemic area for hydatidosis (2–4).

The most affected organs are liver, followed by lungs. Involvement of other organs such as brain, myocardium, muscles, kidneys or bone marrow has been barely reported (2, 4, 5).

Central nervous system (CNS) involvement is rare and occurs in 2–3% of all cases and is more commonly seen in children and young adults (6). Cerebral cysts are usually single, spherical and unilocular. Middle cerebral artery territory is a common location for hydatid cyst; however involvement of orbits, ventricles, hypothalamus, cerebral aqueduct, cavernous sinuses, pons, cerebellum and subarachnoid and extradural spaces has been reported (1, 2, 5–7).

Clinical manifestations may vary based on location and size of the lesions and they can be asymptomatic for years until they grow large enough to evoke symptoms. Symptoms in CNS involvement are often nonspecific including headache, nausea and vomiting, hemiparesis, seizures, altered visual field and gait disorders (8).

Here we report a case of intracranial hydatid cyst presented with headache and visual disturbance, to highlight the importance considering this diagnosis facing nonspecific complaints.

## Case Report

A 12-year-old boy, previously healthy, presented to the Ophthalmology Clinic of Vali-E-Asr Hospital, Birjand, Iran with complaints of vision disturbance and headaches for 2 months. He had no history of headaches until 2 months earlier when he began to develop frontal headaches. Mild and intermittent at first, his headaches progressively got more severe and constant. He had received no medication nor had he undergone any evaluation. On physical examination bilateral papilledema was detected; however other neurological exams were normal.

**Fig. 1:**
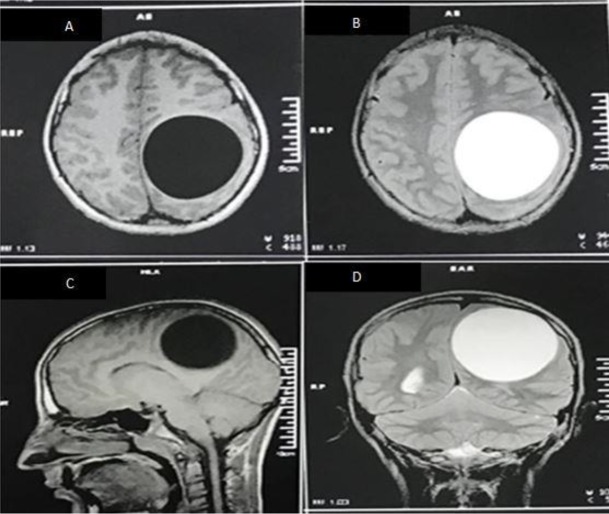
Patient’s Brain MRI A) T1W sequence in axial view, shows a hypo intense oval shaped intraparenchymal cystic lesion with distinct borders in the left parietal lobe B) T2W sequence in axial view, a hyper intense oval shaped intra-parenchymal cystic lesion C) Sagittal view of T1W sequence D) Coronal view of T2W sequence (Original images)

Patient was admitted to pediatric ward for more evaluation. Complete blood count (CBC) showed a leukocyte count of 13600 /mm ^3^ with eosinophilia of 4%. Blood iono-gram and other lab data revealed no significant finding. Brain MRI was performed and showed a large single, oval-shaped, unilocular, intracranial cyst with the diameters of 8 cm in the left parietal lobe with putting mass effect on surrounding structures (Fig. 1).

**Fig. 2:**
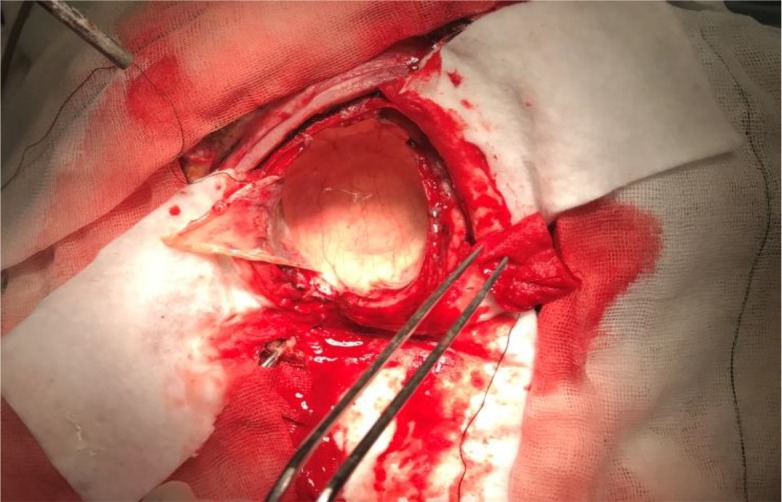
Appearance of the cyst during removal (original image)

**Fig. 3:**
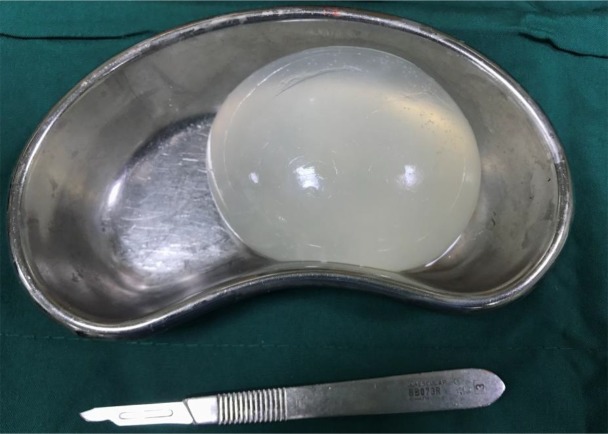
Gross appearance: Total extraction of the hydatid cyst without rupture (original image)

**Fig. 4:**
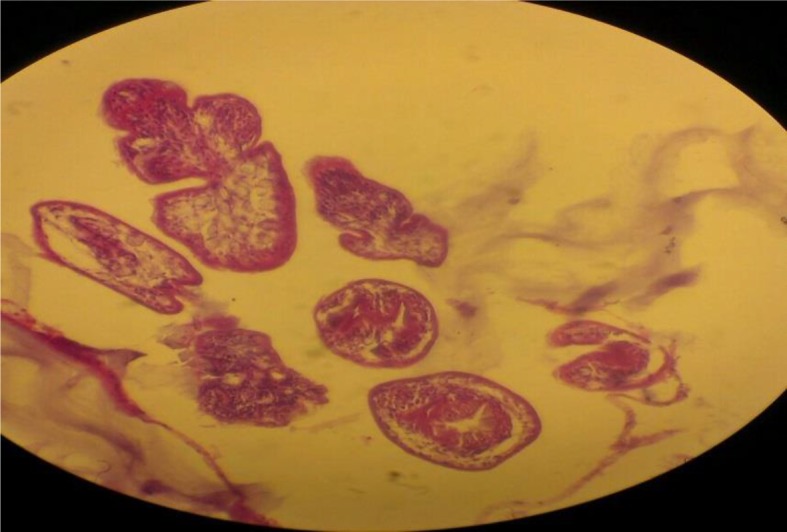
Microscopic view of the specimen in which multiple protoscoleces are seen (original image)

**Fig. 5:**
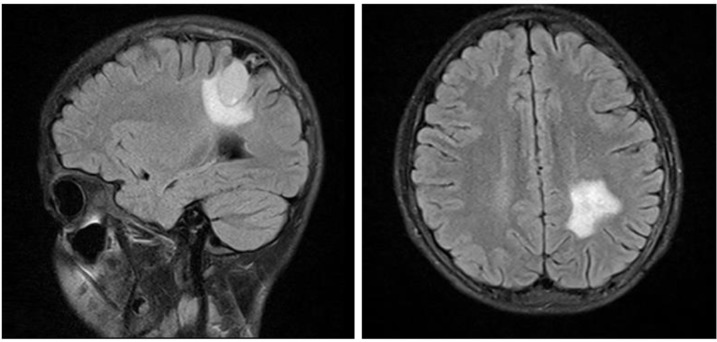
Follow-up brain MRI after one year showing surgery scar and no recurrence (original images)

Investigations for other organ involvement, such as a chest radiograph, an abdominal ultrasonography and an echocardiography were performed but no other infestation was detected. A neurosurgery consultation was requested and the patient was transferred to neurosurgery ward. He underwent surgical exploration, and by using Dowling's technique, the cyst was totally extracted without rupture (Fig. 2 and 3). Specimen was sent for histopathologic examination and the result confirmed the diagnosis of cerebral hydatidosis. Protoscoleces were seen in the specimen (Fig. 4). After the operation, the patient received albendazol (10 mg/kg/bid/d) that was continued for six month postoperatively. In the one-year follow-up, our patient was symptom-free and Brain MRI showed no recurrence (Fig. 5).

It is notable that we obtained confirmation from Research Deputy of Emam Reza Hospital and patient’s agreement to publish her details and images. The study was approved by the university.

## Discussion

Primary intracranial hydatid cyst is an extremely rare condition that is seen in 1–2% of all space occupying intracranial lesions even in endemic areas (9). Most cerebral hydatidosis cases are associated with lesions in the liver, lungs or other organs (10).

Infection usually originates from consuming products related to intermediate hosts (typically sheep) or contaminated water. After ingestion of parasite eggs, they pass intestine mucosa and spread via portal circulation; therefore liver and lungs are considered as most affected organs (1, 2, 6, 9).

Intracranial lesions are expectedly seen in the middle cerebral artery territory as spherical, single, well-defined cysts like what we detected in our patient (1, 2, 7, 11).

Primary intracranial lesions are often found in children and young adults and they are usually asymptomatic until cysts grow large enough to put mass effect on surrounding structures (9).

Size, location and the effect of cyst on surrounding structures specify symptoms. Headache, nausea/vomiting and blurred vision are common neurological presentations that are mainly caused by increased intracranial pressure (ICP) and mass effect (5, 8). Our patient had headache, blurred vision and papilledema that could be attributed t an elevated ICP.

It is important to rule out the other organ involvement in the cases with unusual cyst location like ours. Lungs evaluation by radiography, liver evaluation by sonography and heart evaluation by echocardiography are necessary (5, 6, 12); however in our patient evaluations revealed no other organ involvement.

Plasma Echinococcus Antibody (IgG) test can be useful and demonstrates the immune response, although it can be negative in isolated hydatid cysts, as it was in our patient (6).

Cranial CT scan and MRI can be useful not only for diagnosis of the disease, but also for surgical planning. In CT scan, A typical intracranial hydatid cyst is demonstrated as an intraparenchymal, homogenous, circular lesion with distinct borders. Hydatid fluid density is almost the same as cerebro-spinal fluid (CSF). Peri-lesional edema and contrast enhancement are unusual for an uncomplicated hydatid cyst and usually are in favor of other pathologies such as abscesses or tumors (11).

In brain MRI, hydatid cyst is a hypo intense lesion in T1 weighted images and a hyper intense lesion with a hypo intense rim in T2 weighted images. Peri-lesional edema and fine peripheral enhancement may be seen in presence of active inflammation (11, 13). Other supra-tentorial cystic lesions such as arachnoid cysts, cystic tumors, abscesses and porencephalic cysts should always be considered as differential diagnoses (2, 6, 9).

Surgical removal is the choice for treatment of both symptomatic and asymptomatic lesions. However prevention of cyst rupture during procedure may be challenging especially in cases in which there is significant adhesion between the cyst and the surrounding parenchyma. Our goal in surgery is intact cyst removal without any rupture to prevent anaphylaxis and recurrence, as we did (14).

There is a variety of surgical techniques for hydatid cyst removal but the most widely accepted technique is Dowling-Orlando’s, in which normal saline irrigation is used to release adhesions and safely remove the cyst. Furthermore, several surgical precautions must be considered to decrease surgical complications, including: suitable patient's head position, sufficient craniotomy and corticotomy with adequate cortical dissection and avoidance of any traction on cyst wall (2, 3, 6, 9, 14).

Intra-operative cyst rupture is the most common surgical complication which is associated with dissemination, anaphylactic reaction and mortality. In the situation of cyst rupture, the cyst content must be removed carefully from surgical field and it is recommended that the cavity is irrigated with hypertonic saline (6, 9, 14, 15).

Medical treatment is an important part of hydatidosis management and albendazol is the drug of choice, usually prescribed for months to years. It decreases the risk of anaphylactic reaction and recurrence rate (16).

## Conclusion

Primary intracranial hydatid cyst is rare condition that must be considered as an important differential diagnosis when facing a cystic intracranial lesion along with other cystic brain lesions such as tumors and abscesses, especially in endemic areas. Surgical removal of the cyst without rupture is the choice of treatment however it may be difficult and needs especial technique and experience.
